# Reframing workload saving in LLM-assisted screening: A commentary on Sciurti et al.

**DOI:** 10.1017/rsm.2026.10114

**Published:** 2026-08-27

**Authors:** Honghao Lai, Jiayi Liu, Long Ge, Janne Estill

**Affiliations:** 1 Department of Health Policy and Management, Lanzhou University, China; 2 Evidence-Based Social Science Research Center, Lanzhou University, China; 3 Lanzhou University, China; 4 University of Geneva, Switzerland

Dear Editors

Sciurti and colleagues recently reported in this journal a careful evaluation of three compact large language models, GPT-4o mini, Llama 3.1 8B, and Gemma 2 9B, applied to title and abstract screening across three published systematic reviews on vaccine literacy, antibiotic-resistant *Acinetobacter baumannii*, and vitamin supplementation in COVID-19.^1^ The three corpora differ in size, topic, and prevalence of eligible records, and the authors evaluated each model individually as well as a majority-voting ensemble at predefined rating thresholds. The headline finding, and the one most likely to shape practice, is the estimated workload reduction of 79% to 91% achieved by the ensemble at a 50-rating threshold across the three reviews, while sensitivity remained high. Compact models that run on local hardware at low cost, with savings of this magnitude, would be a meaningful addition to the systematic reviewer’s toolkit.

This figure deserves close reading, however. It is a retrospective summary computed against ground-truth labels that already exist in the published reviews, and it describes the model’s classification behaviour rather than the time a review team will save when deploying the workflow prospectively. The two are related, but they are not the same quantity. Whether the retrospective figure transfers to a real workflow depends on how the team chooses to handle the records the model excluded, and that choice is not visible in the metric itself.

The metric used by Sciurti et al., defined as the proportion of records correctly excluded by the model out of the total, follows the convention proposed by Syriani et al. and is now widely adopted in this literature.^2^ As a property of the classifier, it is well defined and easy to compute. The risk is that, when reported as “workload saving”, it invites a reading it cannot quite support. A practitioner encountering “79% to 91% workload saving” may reasonably infer that, by adopting the workflow, the team will only need to screen the remaining 9% to 21% of citations, on the grounds that the model has correctly removed the rest. The arithmetic of the metric encourages this reading, since saved and remaining work appear to add up to one. Yet the inference rests on a step the metric does not justify, namely the step of treating the model’s excluded set as work that no longer needs to be done.

Systematic reviews are designed to identify, as completely as possible, all studies that meet the eligibility criteria, and methodological guidance treats missing an eligible study as a more serious error than retaining an irrelevant one. Sciurti et al. acknowledge this asymmetry explicitly when they argue that sensitivity should be prioritised and imprecision tolerated. The implication, however, runs further than the design of the prompt. It also constrains how the team must treat the excluded set after the model has spoken. If completeness is to be preserved, exclusions cannot simply be discarded; they have to be either trusted or checked. To realise the estimated saving of 79% to 91% in practice, the review team must accept the residual risk of missed studies that comes attached to it. This risk is quantified by one minus the model’s sensitivity in the corpus at hand. With Sciurti et al.’s ensemble configuration, sensitivity against the original title and abstract decisions ranged from 90.4% to 94.4%, which corresponds to expected false negative rates of roughly 5.6% to 9.6% within the eligible records of each review. Whether such a rate is acceptable is a judgement the metric cannot make for the team. If the team accepts it, the saving figure transfers to practice. If the team does not, the figure does not transfer.

The case in which the team does not accept the residual risk deserves separate attention, because it is here that the gap between the reported metric and real workload becomes visible. When a team requires complete recovery of any false negatives in the excluded set, the model offers no reliable means of locating them: confidence scores and other internal signals were already used to produce the exclusion decision, and tools such as short rationales or supporting excerpts may reduce the time to inspect individual records but cannot themselves identify which records are safe to skip. For a team that will not accept any risk of having missed an eligible study, this means that every excluded record must be re-examined—unless the workflow can partition the excluded set in a way that provides a verifiable guarantee of complete sensitivity within at least part of it. When no such partition is available, the entire excluded set remains in scope for verification.

Once verification covers the full excluded set, the arithmetic of the workload reduction metric breaks down. The work being verified is no longer limited to the small portion the model handled wrongly; it is the entire portion the model handled at all. In the configuration above, that means roughly 80% to 95% of each corpus has to be re-examined in addition to the records the model retained. The time saved at the LLM step is offset by the time spent at the verification step, and the net workload reduction shrinks well below the headline 79% to 91%. In the limiting case, where the team trusts no part of the excluded set without re-examination, the saving approaches zero. The same model, the same prompt, and the same data therefore support very different statements about workload, and the difference is driven entirely by the verification policy the team has adopted.

The link between classifier behaviour and real workload reduction can be made precise by introducing the idea of a verification-exempt subset. This is the part of the excluded set for which the workflow can credibly assert that no eligible study has been missed, either because the subset has been empirically shown to have 100% sensitivity, or because the design provides another form of guarantee. Records outside this subset still require human checking. The larger the verification-exempt subset, the larger the genuine saving, and a workflow that cannot identify any verification-exempt subset at all cannot, strictly speaking, claim a workload reduction beyond what the team is willing to take on as missed-study risk.

Building on this, we suggest a simple verification-aware measure. Let *W_manual_* = *n · t* denote the baseline workload of fully manual screening, where *n* is the total number of records and *t* the time per record. The workload of the LLM-assisted workflow can be written as

$$\begin{array}{r} W_{\mathrm{LLM}}=T_{\mathrm{setup}}+n_{\mathrm{keep}}t_{\mathrm{keep}}+q_{\mathrm{exc}}n_{\mathrm{exc}}t_{\mathrm{verify}} \end{array}$$

Here, *T_setup_* is the one-time cost of deploying the LLM workflow, encompassing prompt development and testing, any calibration runs needed to select the operating threshold, and the time or compute cost of running the model on the full corpus; *n_keep_* and *n_exc_* are the numbers of records retained and excluded, respectively; *q_exc_* is the proportion of excluded records that the team’s pre-specified verification policy designates for human review—a value set at the protocol stage, before the model is run, as a commitment about verification effort rather than a claim about classifier performance; and *t_keep_* and *t_verify_* are the unit times for reviewing a retained record and verifying an excluded record, respectively. Note that *t_verify_* may be shorter than t_keep when LLM-generated confidence scores, brief rationales, or supporting excerpts are available to guide each verification judgment. We therefore propose a new metric, the verification-adjusted workload reduction (VAWR), defined as:

$$\begin{array}{r} \mathrm{VAWR}=1-\frac{W_{\mathrm{LLM}}}{W_{\mathrm{manual}}} \end{array}$$

VAWR is a workload-accounting metric. It takes *q_exc_* as a given input and returns the workload reduction conditional on that verification policy. It does not specify what *q_exc_* should be, nor does it validate whether a chosen verification strategy provides sufficient statistical assurance against false negatives. Those are separate questions that require a sampling design, a sample size justification, and a principled risk threshold.

Three features of this expression are worth noting. First, *q_exc_* is where verification policy enters the calculation, and it can take any value between 0 and 1, allowing a single formula to describe a spectrum of strategies from full trust to full re-screening. Second, when *q_exc_* approaches 0, *T_setup_* is negligible, and *t_keep_* ≈ *t_verify_* ≈ *t*, VAWR approaches *n_exc_*/*n*—the proportion of all records excluded by the model, which is the quantity a prospective team can directly observe. This differs from the conventional workload-saving metric (*TN*/*N*), which counts only correctly excluded records and requires post-hoc access to ground-truth labels. The two quantities coincide only when sensitivity is perfect (FN = 0); when false negatives are present, *n_exc_*/*n* slightly exceeds *TN*/*N* because it credits the workflow for exclusions that were, in retrospect, erroneous. This is precisely the condition under which the trust assumption embedded in *q_exc_* = 0 is consequential: the prospective team cannot distinguish true negatives from false negatives within the excluded set, so the workload they forgo includes both. Third, when *q_exc_* approaches 1, even a classifier with high specificity yields little practical reduction, because the excluded records must be looked at anyway. The metric thereby makes explicit the conditions under which the standard workload saving figure can be trusted as an estimate of real-world time savings.

Applied to the three reviews in Sciurti et al., the metric reveals a wide range of plausible savings depending on which verification policy a team adopts. We focus on the majority-voting configuration at the 50-rating threshold, which the authors recommend as a reasonable default when an optimal threshold is not known in advance. For simplicity, we assume *T_setup_* ≈ 0 and *t_keep_* ≈ *t_verify_* ≈ *t*, so that differences across scenarios are driven entirely by *q_exc_*. The numbers below are recomputed from values reported in their Table 2.

Consider first the conservative position, in which title and abstract decisions made by the original reviewers are taken as the standard of interest, and any sensitivity below 100% triggers full re-screening of the excluded set. Under this policy *q_exc_* = 1 in the VL review (majority-voting sensitivity 90.4%), the AB review (sensitivity 91.3%), and the COVID-19 review (sensitivity 94.4%). VAWR in all three cases drops well below the reported workload savings of 86.1%, 78.8%, and 87.4%. Now consider a more permissive position, in which full-text inclusion is treated as the standard of interest. Sciurti et al. report 100% sensitivity for majority voting at the 50-rating threshold against this reference in all three reviews, so *q_exc_* ≈ 0 and VAWR returns to the neighbourhood of the reported figures. A middle position, such as a 10% random audit of the excluded set (*q_exc_* = 0.10), reduces the verification burden and places VAWR between these two extremes. It is important, however, to distinguish the workload implications of such an audit from its evidentiary value. Setting *q_exc_* = 0.10 determines the VAWR calculation, but the inferential claim that the remaining 90% of exclusions are safe to skip without further checking depends on whether the audit was statistically powered for that purpose. Finding zero false negatives in a 10% sample does not by itself establish an acceptable false-negative rate in the unverified portion unless the sample was sized against a prespecified confidence bound on the false-negative rate. A partial audit therefore functions as a practical verification policy with computable workload implications, but its inferential claims require a sampling design and sample size justification to be stated alongside the VAWR figure.

The same workflow, then, supports very different statements about workload reduction depending on a choice that has nothing to do with model behaviour. We do not read this as a flaw of Sciurti et al.’s analysis, since their numbers are correct given the metric they used. We read it as a sign that the metric itself needs a companion piece of information, namely the verification policy under which it is to be interpreted.

A second observation arises from the full-text comparison itself. In all three reviews, several configurations achieved 100% sensitivity against full-text inclusion, which means that, in retrospect, no truly eligible study was lost in the LLM exclusions for these corpora. The result is encouraging but retrospective. A reviewer running a new review prospectively cannot know in advance whether such full-text-level sensitivity will hold for their corpus, since it depends on properties of the literature that are only revealed at the full-text stage. Prudent practice will assume it does not until evidence accrues. VAWR is meant to capture this prospective uncertainty by making the verification policy, rather than a hoped-for retrospective property, the basis on which workload claims are made.

Several practical implications follow. For review teams, *q_exc_* should be specified at the protocol stage as part of the verification policy, alongside the screening method and conflict-resolution rules. Several considerations can inform this pre-specification. The primary driver is the acceptable false-negative rate for the review in question: a Cochrane review aiming for comprehensive evidence synthesis may warrant lower tolerance for missed studies—and therefore a higher *q_exc_*—than a rapid review supporting a time-sensitive policy decision. Where published validation data exist for a similar LLM configuration and corpus type, the observed sensitivity in those studies provides an empirical anchor for estimating the residual false-negative rate at a given *q_exc_*. Team size and reviewer experience enter as practical constraints rather than as risk parameters: a larger or more experienced team may be able to sustain a higher verification burden at lower per-record cost, affecting the feasibility of different *q_exc_* choices without changing the acceptable risk threshold itself. The time parameters *t_keep_* and *t_verify_* can be estimated from pilot data or from published benchmarks, which typically report title-and-abstract screening at 5–40 seconds per record; *t_verify_* may be shorter when LLM-generated outputs are available to guide the check. Stating in advance whether the team will accept LLM exclusions, audit a sample of them, or re-screen the full set turns workload saving from a property reported after the fact into a quantity that can be planned for, and it allows reviewers, peer reviewers, and editors to assess whether the chosen policy is appropriate to the question at hand. It is also worth distinguishing between two uses of *q_exc_*: as a prospective policy commitment, in which the team prespecifies the verification fraction before running the model; and as an empirically informed quantity, in which the verification fraction is calibrated by the results of a validation study. In de novo reviews, only the first use is available in advance. The second requires a powered audit and a stated confidence bound on the false-negative rate in the unverified portion; its inferential implications are a separate question from the VAWR calculation and should be reported alongside it rather than folded into it.

For developers of LLM-assisted screening systems, lowering *q_exc_* may be more valuable than further pushing classifier sensitivity.^3^ Calibrated confidence outputs that flag low-confidence exclusions for human review, abstention mechanisms that route ambiguous records to an “uncertain” bucket rather than to the excluded set, and risk stratification of the excluded set are all design choices that shrink the part of the workflow needing verification without compromising completeness. Each of these moves a portion of the excluded set into a verification-exempt subset that can be defended on grounds other than blanket trust. Reducing *T_setup_* is a complementary design goal. Workflows that require extensive prompt engineering, threshold calibration runs, or large pilot sets before deployment raise the setup cost term in VAWR, which erodes the net workload reduction particularly for smaller reviews where the corpus may not be large enough to amortise the initial investment. Streamlined deployment pipelines and validated default configurations that transfer across corpus types would widen the class of reviews for which LLM-assisted screening offers a genuine net saving.

More broadly, we suggest that workload saving be reported together with the pre-specified verification policy, the values of *n_keep_* and *n_exc_*, an estimate or bound on *q_exc_* under that policy, and—where *q_exc_* is derived from a sample audit rather than a blanket policy commitment—the statistical basis for that estimate: the sampling design, the achieved sample size, and the confidence interval on the false-negative rate in the unverified portion of the excluded set. None of this requires new infrastructure, and it would sit comfortably alongside the reporting items already promoted by recent guidance on AI in evidence synthesis,^4^ where it could plausibly feature in future updates as the verification dimension of workload reporting.

## Data Availability

No new data were generated or analysed in support of this research.
